# Global Health Mentoring Toolkits: A Scoping Review Relevant for Low- and Middle-Income Country Institutions

**DOI:** 10.4269/ajtmh.18-0563

**Published:** 2018-11-14

**Authors:** Bhakti Hansoti, Anna Kalbarczyk, Mina C. Hosseinipour, Dorairaj Prabhakaran, Joseph D. Tucker, Jean Nachega, Lee Wallis, Jonathan K. Stiles, Adriane Wynn, Chelsea Morroni

**Affiliations:** 1 Department of Emergency Medicine, Johns Hopkins School of Medicine, Baltimore, Maryland;; 2 Department of International Health, Johns Hopkins School of Public Health, Baltimore, Maryland;; 3 Johns Hopkins Center for Global Health, Johns Hopkins University, Baltimore, Maryland;; 4 University of North Carolina Project, Lilongwe, Malawi;; 5 Department of Medicine, University of North Carolina at Chapel Hill School of Medicine, Chapel Hill, North Carolina;; 6 Public Health Foundation, Delhi, India;; 7 Centre for Chronic Disease Control, Delhi, India;; 8 Faculty of Infectious and Tropical Diseases, London School of Hygiene and Tropical Medicine, London, United Kingdom;; 9 UNC Project-China, Guangzhou, China;; 10 Departments of Epidemiology, Infectious Diseases and Microbiology, University of Pittsburgh Graduate School of Public of Health, Pittsburgh, Pennsylvania;; 11 Department of Medicine and Centre for Infectious Diseases, Stellenbosch University Faculty of Medicine and Health Sciences, Cape Town, South Africa;; 12 Departments of Epidemiology and International Health, Johns Hopkins Bloomberg School of Public Health, Baltimore, Maryland;; 13 Division of Emergency Medicine, University of Cape Town, Cape Town, South Africa;; 14 Division of Emergency Medicine, Stellenbosch University, Stellenbosch, South Africa;; 15 Department of Microbiology Biochemistry and Immunology, Morehouse School of Medicine, Atlanta, Georgia;; 16 Division of Global Public Health, Department of Medicine, University of California San Diego, San Diego, California;; 17 University of California Global Health Institute, San Francisco, California;; 18 Department of International Public Health, Liverpool School of Tropical Medicine, Liverpool, United Kingdom;; 19 Botswana UPenn Partnership, Gaborone, Botswana;; 20 Botswana Harvard AIDS Institute Partnership, Gaborone, Botswana;; 21 Wits Reproductive Health and HIV Institute, University of Witwatersrand, Johannesburg, South Africa;; 22 Women’s Health Research Unit, School of Public Health and Family Medicine, University of Cape Town, Cape Town, South Africa

## Abstract

Capacity building in low- and middle-income country (LMIC) institutions hinges on the delivery of effective mentorship. This study presents an overview of mentorship toolkits applicable to LMIC institutions identified through a scoping review. A scoping review approach was used to 1) map the extent, range, and nature of mentorship resources and tools available and 2) to identify knowledge gaps in the current literature. To identify toolkits, we collected and analyzed data provided online that met the following criteria: written in English and from organizations and individuals involved in global health mentoring. We searched electronic databases, including PubMed, Web of Science, and Google Scholar, and Google search engine. Once toolkits were identified, we extracted the available tools and mapped them to pre-identified global health competencies. Only three of the 18 identified toolkits were developed specifically for the LMIC context. Most toolkits focused on individual mentor–mentee relationships. Most focused on the domains of communication and professional development. Fewer toolkits focused on ethics, overcoming resource limitations, and fostering institutional change. No toolkits discussed strategies for group mentoring or how to adapt existing tools to a local context. There is a paucity of mentoring resources specifically designed for LMIC settings. We identified several toolkits that focus on aspects of individual mentor–mentee relationships that could be adapted to local contexts. Future work should focus on adaptation and the development of tools to support institutional change and capacity building for mentoring.

## Introduction

Capacity building in global health is contingent on the delivery of advance mentoring in low- and middle-income country (LMIC) institutions. Mentorship here is defined as providing support and training for personal and professional development related to global health practice, education, and research. Mentorship not only has the ability to build institutional capacity, but also fosters long-lasting relationships for collaboration. Although many academics and institutions in LMIC settings receive formal or informal mentorship, the specific challenges for effective mentorship are less well addressed. Toolkits provide practical guidance and the structure to support effective mentor–mentee relationship.

Challenges to effective mentorship can be considered at the mentee, mentor, and institutional levels. At the mentee level, differences in cultural, social, and economic backgrounds between mentors and mentees may constrain the success of the mentoring relationship.^1^ For example, cultural etiquettes in social standing, gender norms, and respect may impact effective and transparent communication.^2^ Mentors may adopt a paternalistic view of the mentorship relationship, which may limit the mentees’ ability to achieve full independence.^3^ At the mentor level, investigators in LMIC settings have far fewer opportunities for mentor training compared with investigators in high-income countries (HICs). Hence, many relationships may veer away from mentorship toward taking advantage of mentees because of economic realities, institutional pressures, and social norms.

Mentoring relationships in LMIC institutions are further faced with several well-documented challenges related to equity compared with institutions in HICs. For example, LMIC institutions may be less likely to foster a culture of mentorship and collaboration building, and may have fewer opportunities for training, career advancement, and recognition.^4^ Institutional capacity to develop and support mentoring is also challenged by complexities of funding and support for research and innovation at the health systems level. For example, there is often a lack of grant funding, or higher education platforms and innovation hubs at the national level.^5^ Furthermore, because of “brain drain” and related effects, LMIC settings may have a limited number of experienced mentors who may be overwhelmed with mentees’ diverse needs.

The use of toolkits is a potential mechanism for providing support to mentees in a systematic way. Toolkits can help focus mentors and mentees activities together, and provide a framework to define goals and milestones. Toolkits are resources that may be written or online products that provide activities, checklists, and suggestions. In the context of mentorship, they provide a systematic approach to guide the mentoring relationship and often include milestones for evaluation and refocusing. We conducted a scoping review to identify and examine the toolkits currently available to support LMIC mentorship in global health.

## Methodology

We chose a scoping review approach to 1) map the extent, range, and nature of mentorship resources and tools available and 2) to identify knowledge gaps in the current literature. We used Arksey and O’Malley’s framework for conducting this scoping review, which aims to summarize the current evidence, best practice guidelines, and identify areas of limited understanding.^6^ Unlike a formal systematic review, scoping reviews do not use a priori inclusion criteria and do not assess the quality of published articles included in the review.

To identify toolkits, we collected and analyzed data provided online that met the following criteria: written in English and from organizations, and individuals involved in global health mentoring. We searched electronic databases, including PubMed, Web of Science, and Google Scholar, and Google search engine. Search terms included “mentor” or “mentorship,” “global,” or “international,” and “tool,” “guide,” or “manual.” Search results were analyzed for content pertaining to mentorship in global health and/or toolkits. In addition, we reviewed the titles and tables of identified toolkits and included those that provided guidance to mentors, mentees, or institutions related to developing, fostering, and/or sustaining mentorship relationships in a global health setting. We did not limit findings to a specific time period. The online search was supplemented by informal solicitation for additional resources, by email, to the American Society of Tropical Medicine and Hygiene (ASTMH) special issue authorship group.

Any resources that provided guidelines on supporting mentoring relationships and building mentorship capacity that could be applied to an LMIC setting (as decided on by authors B. H., A. K., and A. W.) were included in this review. The following information was extracted from each identified resource: title, description, intended user, global health competencies addressed, tool type (e.g., formative, descriptive, evaluation), author(s), institution, and weblink. We also identified whether the tool was “descriptive” and described good/effective mentoring relationships, “formative” and provided information on how to execute a good/effective mentoring relationships, or “evaluative” and provided guidance on how to evaluate the success of these mentoring relationships and programs. Mentoring competencies have been framed previously by Hamer et al. and Flemming et al., in the following categories: Maintaining effective communication (COM); aligning expectations (EXPs); assessing understanding (UND); addressing diversity; promoting professional development; fostering independence; professional integrity and ethical conduct; overcoming resource limitations; fostering institutional change (CHA).^7–10^ The Hamer et al. study further delineated the skills required to attain these competencies.^10^

## Results

A total of 18 toolkits were judged relevant to this scoping review (Table 1). We excluded several guides that focused solely on mentoring junior faculty at U.S. institutions or staff at the National Institutes of Health (NIH). Few toolkits were focused specifically on mentoring in LMIC setting (*n* = 3)^11–13^ with the majority published by North American organizations (*n* = 15) (Table 1). Seven toolkits were created by universities,^13–19^ six by professional organizations,^20–25^ one by a funding agency,^12^ two by training consortia,^11,26^ and two by journals.^8,27^ Many were focused on mentoring specifically in the health sciences (*n* = 11). All were relevant for mentoring in global health, but six specifically targeted mentoring in a global health context. Several included checklists and predeparture training guidelines that focus on the HIC mentee experience in an LMIC setting, three of which were included in this scoping review.^19,22,23^

**Table 1:** Mentorship toolkits, intended users, competencies, formal, tools identified, and source

Name of toolkit	Description	Intended user (mentee/mentor/institution)	Competencies addressed	Format (evaluative/formative/descriptive)	Tools	Author, institution	Weblink
Mentoring handbook^11^	Guide for mentees and mentors on the benefits of mentorship and building a relationship.	Mentors and mentees	COM, EXP, UND, and PRO	Descriptive		The Afya Bora Fellowship in Global Health Leadership	http://www.afyaboraconsortium.org/materials/Mentoring%20Handbook.pdf
	Distinguishes types of mentorship and discusses benefits of mentorship to both mentors and mentees					Category: Training consortia	
Fogarty Fellows Toolkit for Mentorship^12^	Short PowerPoint presentation that provides steps toward institutionalization of mentorship including 1) Assess needs, 2) develop tools, 3) provide training, and 4) integrate across levels. Differentiates between types of mentors–primary vs. thematic.	Institutions and mentors	DIV, PRO, RES, ETH, and CHA	Descriptive	Links to external resources	Fogarty	http://fogartyfellows.org/wp-content/uploads/2015/01/8DZunt.pdf
						Category: funding agency	
Morehouse SOM: Mentoring Academy^13^	Includes mentoring tools we gathered from the Malaria Capacity Development Consortium for mentoring LMIC’s. Explores team mentoring and peer mentoring approaches.	Mentors and mentees	COM, EXP, UND, ETH, CHA, DIV, and PRO	Has both formative and evaluative tools		Morehouse School of Medicine	http://www.msm.edu/Research/research_centersandinstitutes/RCMI/MentoringAcademy/AboutMA.php
						Category: University	
Faculty Mentoring^14^	Includes a list of 10 topics that may be covered with academic mentoring with a focus on obtaining promotions, managing time and work-life.	Mentees	COM	Descriptive	N/A	Stanford University, School of Medicine	https://med.stanford.edu/academicaffairs/professoriate/FacultyResources/counseling/mentoring.html
						Category: University	
Mentorship Guidelines for Global Health Mentors^15^	Succinct document that differentiates between types of mentors: advising, career mentoring, and project mentoring.	Mentor; doesn’t differentiate between LMIC or HIC mentee.	COM, EXP, PRO, and IND	Formative and descriptive	N/A	UCSF	https://www.aap.org/en-us/Documents/soich_gh_international_faculty_ucsf.pdf
						Category: University	
Guidelines for mentor/mentee conversations^16^	A document with guidelines on conversations between mentor/mentee on the tenure and clinician educator tracks	Mentors	COM	Descriptive	Conversation guidelines and coaching tips; letter templates; links to external resources	Perlman School of Medicine	https://www.med.upenn.edu/fapd/docurepo/guidelines-for-mentor-mentee-conversations.html
						Category: University	
Mentoring Program Virtual Binder^17^	Guides for faculty on how to establish mentoring relationships, postdoctoral fellows mentoring standards, sample individual development plan, mentor orientation and guidelines, reference letters	Mentors	COM, EXP, UND, PRO, and ETH	Includes all three	Mentoring standards; letter templates; faculty guidelines; orientation and guidelines; links to external resources	CFAR UCSF	http://cfar.ucsf.edu/mentoring/virtual-binder
						Category: University	
University of Washington Mentoring resources^18^	Specific tools that can be adapted such as sample mentoring agreements and evaluation forms.	Mentors	EXP and UND	Evaluative	Mentoring guide; mentoring agreements; evaluation forms; recognition forms; links to external resources.	University of Washington	https://www.washington.edu/medicine/pediatrics/pednet/mentoring
						Category: University	
Preparing for International Health Experiences: A practical guide^19^	Book for medical trainees preparing to work overseas. Provides information on legalities, cultural and language competencies, and ethical issues. Multiple specialities are covered and provide accompanying online toolkits.	Mentee; HIC traveling to LMIC	EXP and UND	Formative		Edited by Akshaya Neil Arya	https://www.routledge.com/Preparing-for-International-Health-Experiences-A-Practical-Guide/Arya/p/book/9781138627277
						Category: University	
American Heart Association Mentoring Handbook^20^	A lengthy guide that covers topics related to the forming, maintaining, and concluding mentoring relationships from the perspective of the mentor, mentee, and mentoring environment. Several chapters are focused on cardiovascular science, one chapter is focused on women, underrepresentived minorities, and those who have been trained abroad; and another on foreign medical school graduates.	Mentors and mentees	COM, DIV, ETH, EXP, IND, RES, and UND	Formative and descriptive	N/A	American Heart Association	http://my.americanheart.org/idc/groups/ahamah-public/@wcm/@sop/documents/downloadable/ucm_319794.pdf
						Category: Membership Association	
CCGHR—“Mentoring Modules”^21^	6 downloadable modules for mentors and establishing a mentorship program. Modules provide tools for mentors to complete.	Mentors	IND and PRO	Formative		CCGHR	http://www.ccghr.ca/resources/mentorship-and-leadership/mentoring-modules/
						Category: Membership assoc.	
CUGH Field Experience Checklist^22^	Checklist for students preparing for an international health experience. Compilation of questions to ask oneself (scroll to bottom of webpage)	Mentee; HIC traveling to LMIC.	EXP and UND	Formative		CUGH	https://www.cugh.org/content/annotated-list-online-global-health-resources
						Category: Membership assoc.	
Faculty Preparation for Global Experiences Toolkit^23^	Discusses considerations for international exchanges and collaborations. Provides an overview of the faculty role in preparing a student to travel/live/work overseas; this includes a section on “debriefing.”	Mentors preparing HIC students for international travel	EXP and COM	Formative		National League for Nursing	http://www.nln.org/docs/default-source/default-document-library/toolkit_facprepglobexp5a3fb25c78366c709642ff00005f0421.pdf
						Category: Membership assoc.	
Getting the Most out of Your Mentoring Relationship (available for members)^24^	An online resource that provides links to webinars, workshops and articles related to developing and maintaining mentorship relationships and programs.	Institutions, Mentors and Mentees	COM, ETH, and PRO	Formative and Descriptive	Webinars: Building a Mentoring Program, Interdisciplinary Mentoring; Workshops: The Importance of Mentoring Relationships; Articles: Sponsorship, Interdisciplinary Mentoring, Coaching, Mentoring Circles.	Association for Women in Science	https://awis.site-ym.com/?Mentoring
						Category: Membership Association	
Mentoring and Being Mentored^25^	Includes sections on: How to Find a Mentor, General Mentoring Strategies, How to be a Good Mentor or Protégé, Transitioning from Protégé to Mentor. Within each section are articles and presentations related to the topic.	Mentors and Mentees	COM, EXP, PRO, and IND	Formative and descriptive	(This site includes a lot of links to articles and presentations.)	American Physiological Society	http://www.the-aps.org/mm/Careers/Mentor/Mentoring-and-Being-Mentored
						Format: Membership Association	
I-TECH Clinical Mentorship^26^	Multiple tools and guidance documents. Focuses on clinical mentoring for HIV/AIDS care. Includes different models of mentoring, steps in the mentoring process, training clinical mentors, and monitoring and evaluating mentoring programs.	Institutions and mentors	COM and UND	Includes all three	Document guidelines for mentorship models, setting up and managing mentoring programs; case studies; participant workbooks; training curricula; M&E tools; Image Library	I-TECH,	https://www.go2itech.org/HTML/CM08/toolkit/overview/index.html
						Category: Training consortia	
Nature’s guide for mentors^27^	Establishing a definition of good scientific mentoring drawing on evidence from Nature's awards for creative mentoring in science	Mentors and Mentees	COM, EXP, and UND	Descriptive		Nature	https://www.nature.com/articles/447791a
						Category: Journal	
Medical Student Mentoring Guide^28^	Resource for faculty that mentor medical students, which consists of a set of tools to guide mentors and provides a framework for approaching mentor–mentee interactions	Mentors	COM	Formative	Mentoring grid structured around common mentoring domains, Mentoring prompts that are designed to stimulate discussion and guide conversations, and a PowerPoint presentation that provides an overview and introduction to the value and role of mentors in medical education.	American Association of Medical Colleges	https://www.mededportal.org/publication/9861/
						Category: Journal	

CFAR UCSF = Center for AIDS Research, University of California, San Francisco; CHA = fostering institutional change; COM = maintaining effective communication; DIV = addressing diversity; ETH = professional integrity and ethical conduct; EXPs = aligning expectations; HIC = high-income countrie; IND = fostering independence; LMIC = low- and middle-income country; PRO = promoting professional development; RES = overcoming resource limitations; UND = assessing understanding.

Most toolkits were intended for mentors and provided guidelines on how to effectively set expectations, communicate with mentees, and build the mentoring relationship (*n* = 14). Ten toolkits were focused on guiding the mentee, and provided checklists or outlined important “to-do’s” to prepare for and have a successful experience. Only three resources specifically targeted the institution’s role in mentorship and provided recommendations for institutionalizing and supporting mentorship. Tools were either descriptive (*n* = 5), formative (*n* = 5), or both (*n* = 4). Only four tools provided guidance on how to evaluate the success of mentoring relationships and programs.

### Institution–mentor relationship.

Three tools focused on developing mentorship programs within institutions. They included guidelines on setting up and managing programs and one provided evaluation forms. The I-TECH clinical mentorship toolkits provided specific guidance on how to build clinical mentoring capacity at the institution.^26^ As such, these toolkits provided resources to cultivate effective mentoring relationships from the mentor prospective and provided guidelines on how to train mentors. This toolkit is focused on developing and managing mentorship programs using a case-based approach. The Fogarty toolkit on “integration and institutionalization of mentorship training” is a descriptive tool that presents a stepwise approach to building institutional mentorship capacity.^12^ The Association for Women in Science’s guide also includes content for institutions, including webinars on how to build a mentoring program.^24^

### Mentor–mentee relationship.

Many toolkits (*n* = 8) discussed the roles that different mentors can have on professional development. A variety of tools were provided, including codes of conduct/practice, mentorship agreements, letter templates, and guides for individual development plans. These toolkits provided resources for evaluation and monitoring of the mentor–mentee relationship.^13,17,18^ The Malaria Capacity Development Consortium (MCDC) provided a strong example of how mentoring could be conducted in a LMIC context.^28^ The aim of the MCDC mentoring program is to help mentees with their personal and professional development, so that they are able to reach their full potential as self-reliant, self-confident, and independent scientific researchers. The MCDC also provided a code of conduct, commandments for good mentoring, examples of agreements, and evaluation forms that allow the mentee to document, manage, and record the mentoring meetings.

### Competencies.

Many of the toolkits addressed three central competencies: COM (*n* = 13), EXPs (*n* = 11), and UND (*n* = 9). These competencies were achieved by presenting specific information on how to communicate with mentees, provide feedback, and establish goals and expertise. Several toolkits mentioned how a mentor can assist in nurturing independence (*n* = 4) and professional development (*n* = 8). Only five mentioned ethics and professionalism, including helping the mentee learn about requirements for authorship and the importance of different authorship positions. Overcoming resource limitations was mentioned by two toolkits and CHA was also included in two toolkits.

## Discussion

Successful global health research and practice is dependent on strong mentorship, which requires training, guidelines, and standards for mentees, mentors, and mentoring institutions. We undertook this scoping review to identify and evaluate currently available mentoring toolkits. Most of the toolkits identified by the scoping review were developed by organizations in North America. Overall, there is a dearth of resources created by LMICs. In particular, few tools focused on how to support mentoring at the institutional level and few focused on additional competencies (e.g., ethics, resource limitations, CHA). Furthermore, none of the resources published from HIC authors and institutions included information on how they may be adapted to various settings, including LMIC settings.

Most toolkits focused on the individual mentor–mentee relationship. In particular, a number of toolkits provided standardized approaches to evaluate the training outcomes of mentees. Toolkits that focused on the mentee generally highlighted the importance of individualized development plan or similar documents developed together by the mentor and mentee. Many of the guidance documents recommend early engagement with the mentor to help establish rapport, define explicit short- and long-term goals, and clarify mutual expectations. In addition to established toolkits, it is also appreciated that mentors may use academic outputs to monitor trainee progress. For example, the Southern Africa Consortium for Research Excellence uses a logbook as a PhD mentoring tool.^29^

We identified no toolkit that provided specific guidance on group mentorship activities. Group mentorship activities allow for peer mentoring and for mentees to discover a wide range of skillsets and training activities, while facilitating peer-to-peer mentoring. In the absence of formal guidelines for group mentorship activities, some research institutions may develop their own peer-training opportunities, such as “Work in Progress” meetings, where trainees and faculty meet to present and discuss current research of the trainees.^30^ This forum provides opportunities for scientific presentation, peer review, and troubleshooting of research challenges, drawing on the experiences of a collective group. Regular English language journal clubs may also be useful. Bringing together mentees at similar career stages for directed activities contributes greatly to achieving milestones. For example, a dedicated writing retreat with those actively working on a manuscript provides motivation for completion and an available audience for peer review of the work. In particular, in a research-constrained environment, where the number of mentors are few, group mentorship may provide a way to overcome limitations and build a supportive community for trainees to facilitate the exchange of ideas to overcome resource limitations and specific institutional challenges.

There are also fewer resources available for mentors to learn about the art and science of mentorship in LMIC settings. From the perspective of mentors, mentorship is positioned within a broader landscape of responsibilities. Competing agendas from teaching, clinical and research obligations sometimes interfere with training goals at the site. Mentoring foreign trainees can include overcoming time-consuming and often necessary bureaucratic hurdles such as regulatory approvals, resident permits, and ensuring safety. A successful intercountry mentor–mentee relationship often goes beyond a professional relationship and includes developing rapport with the family, providing personal support (e.g., advice about schools or childcare), and helping accompanying spouses.

Acknowledging the need for well-trained mentors in LMICs, The University of California Global Health Institute has worked in partnership with global health leaders to hold “Mentoring the Mentors” workshops adapted regionally in South America (2013), East Africa (2013), Southern Africa (2016), South Asia (2015), and most recently West and Central Africa (2018). The workshops strived to cover everything from goal setting to funding mentoring activities.^31^

There is little literature focused on how to initiate institutional changes with respect to mentorship practices. This gap may be related to a weaker evidence base supporting this important aspect of mentorship. Institutions in both LMICs and HICs tend to recognize research contributions more than research training contributions. Institutional leaders must be convinced that they will generate a return on their investment if they invest in their mentors and mentees.

Examples of institutional capacity-building models for mentorship include that of the Mentoring Academy at the Morehouse School of Medicine (MSM), which is supported by the NIH Research Centers in Minority Institutions, and the African regional training workshops conducted by the Fogarty International Center (FIC)-supported Global Health Program for Fellows and Scholars consortia described earlier. In addition, the NIH-funded National Research Mentoring Network^13^ and the Building Infrastructure Leading to Diversity programs in the United States conduct regional training to enhance biomedical, clinical, and translational research mentoring in U.S. institutions.^13^ Some of the key lessons learned from these programs that could be applied to global health research conducted in LMICs include the need for a sound argument based on return on investment at the institutional leadership level; the need to demonstrate through well-designed institutional self-study, the benefits gained by the institution by systematizing mentoring of mentees and showing better outcomes among those receiving effective mentoring and those who did not; and the need to train faculty and learners to imagine the mentor–mentee relationship not only as a capacity building and development initiative, but also a succession planning activity to ensure continued advancement of health for the country.

### Limitations.

This scoping review has several limitations. First, toolkits are only one way to strengthen global health mentorship in LMICs. These toolkits are not all-inclusive and have their own design flaws, omissions, and assumptions. We did not formally evaluate the quality of toolkits. Second, there was relatively less material created by LMIC mentors for LMIC mentees, potentially because of not being reached by our online search process. This likely represents the largest component of global health training and requires more careful attention. The adoption of best mentoring practices from developed country settings into LMICs is not merely a straightforward issue of uptake and implementation but instead a complex process of adaptation into different academic and cultural settings, and merging with local practices.

## Conclusion

Mentorship in global health research and practice is complex, but crucial to effective studies and programs. The toolkits provided in this scoping review provide practical advice for mentorship and can be used to spur change. Future endeavors in this field should seek to develop guidance around group mentoring activities and how to successfully adapt existing mentorship tools to diverse LMIC contexts.
